# A Case Report on Cervicofacial Subcutaneous Emphysema and Its Management: A Unique Presentation of Congenital Defect of the Foramen of Huschke

**DOI:** 10.7759/cureus.47078

**Published:** 2023-10-15

**Authors:** Siti Nurafiqah Sharudin, Nor Azirah Salahuddin, Siti Asmat Md Arepen, Noor Azrin Md Anuar, Nor Eyzawiah Hassan, Shahrul Hitam, Fadzilah Ismail, Salina Husain

**Affiliations:** 1 Otorhinolaryngology - Head and Neck Surgery, Faculty of Medicine, Hospital Canselor Tuanku Muhriz, Universiti Kebangsaan Malaysia, Cheras, MYS; 2 Otorhinolaryngology - Head and Neck Surgery, Universiti Sains Islam Malaysia, Seremban, MYS; 3 Otorhinolaryngology - Head and Neck Surgery, Hospital Ampang, Ampang, MYS; 4 Otorhinolaryngology, Hospital Sungai Buloh, Sungai Buloh, MYS; 5 Otorhinolaryngology - Head and Neck Surgery, Faculty of Medicine, Hospital Canselor Tuanku Muhriz, Universiti Kebangsaan Malaysia, Kuala Lumpur, MYS

**Keywords:** cervicofacial, emphysema subcutaneous, temporomandibular joint prolapse, persistent foramen hushke, congenital facial defect

## Abstract

Congenital defect to the foramen of Huschke with a manifestation as temporomandibular joint (TMJ) soft tissue herniation in a radiological study is rare. The patient may present with nonspecific symptoms such as otalgia, mandibular joint pain, tinnitus and conductive hearing loss, and scarcely cervicofacial subcutaneous emphysema. Here, we report a patient presented with cervicofacial subcutaneous emphysema secondary to a congenital defect of the foramen of Huschke. A 45-year-old gentleman presented with right-sided neck swelling and right otalgia with a crackling sound over the right ear upon chewing. Examination shows right-sided fullness with subcutaneous emphysema from the zygoma to the upper neck. Otherwise, it is non-tender with no skin changes, and the facial nerve is intact. Otoendoscopy shows erythematous soft tissue bulging of the anterior wall of the right external auditory canal (EAC) upon closing the mouth and prolapsing upon mouth opening. The right tympanic membrane was intact. Contrast-enhanced computer tomography (CECT) of the neck and temporal region revealed extensive cervicofacial subcutaneous emphysema with a bony defect at the anterior wall of the right EAC, indicating fistulous communication between the right EAC and TMJ. The subcutaneous emphysema resolved on the treatment of the right otitis externa. The patient is subjected to a combined approach of open and endoscopic-assisted repair of the anterior EAC wall defect. TMJ herniation into the anterior EAC is rare; however, the patient presentation may vary. CECT is the gold standard for diagnosing and facilitating treatment options. Treatment choice is based on the patient's condition, including conservative or surgical intervention.

## Introduction

The tympanic bone is in the form of a U-shape at birth, which has anterior and posterior prominence and is usually closed by the age of five. However, defective membranous ossification leads to congenital anterior wall defect, which also been called foramen of Huschke [1]. This persistent defect may permit retrodiscal soft tissues of the temporomandibular joint (TMJ) to herniate into the ear canal [2]. Congenital defect to the foramen of Huschke is rare, with only 0.4% manifesting as TMJ soft tissue herniation on a radiological study [1]. The patient may present with nonspecific symptoms such as otalgia, mandibular joint pain, tinnitus and conductive hearing loss, and scarcely cervicofacial subcutaneous emphysema. Here, we report a patient presented with cervicofacial subcutaneous emphysema secondary to a congenital defect of the foramen of Huschke.

## Case presentation

A 45-year-old Malay gentleman presented with right-sided facial swelling for a three-day duration associated with throbbing pain, especially when he tries to eat. He noticed there was a cracking sound over the right ear upon chewing. He had a history of right otitis external with impacted earwax two weeks prior, which was treated with oral cefuroxime and ciprofloxacin ear drops for one week. The patient had neither a history of trauma nor surgery that could have caused the condition; however, he experienced multiple episodes of TMJ subluxation, which resolved with self-manual manipulation. 

**Figure 1:**
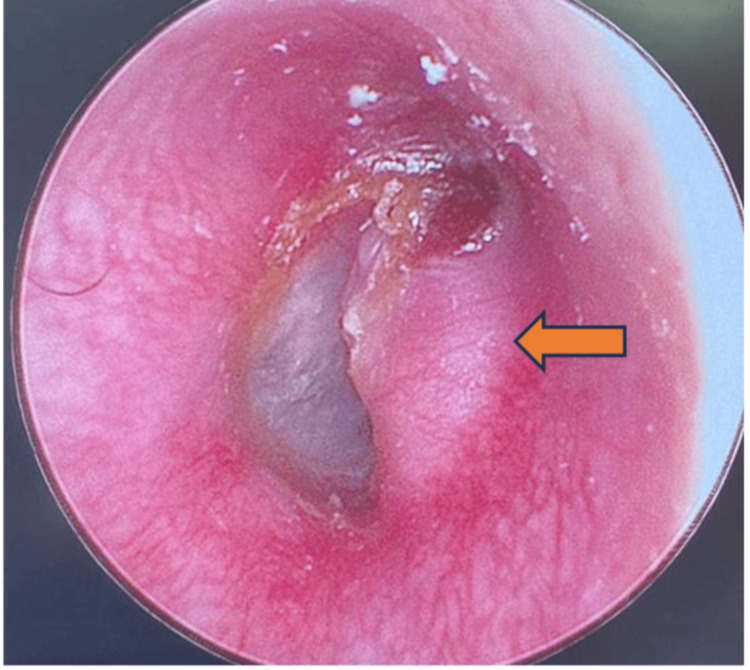
Otoscopic examination of the right ear shows soft tissue protrusion at the anterior wall of EAC close to the TMJ region upon closing the mouth (arrow).

**Figure 2:**
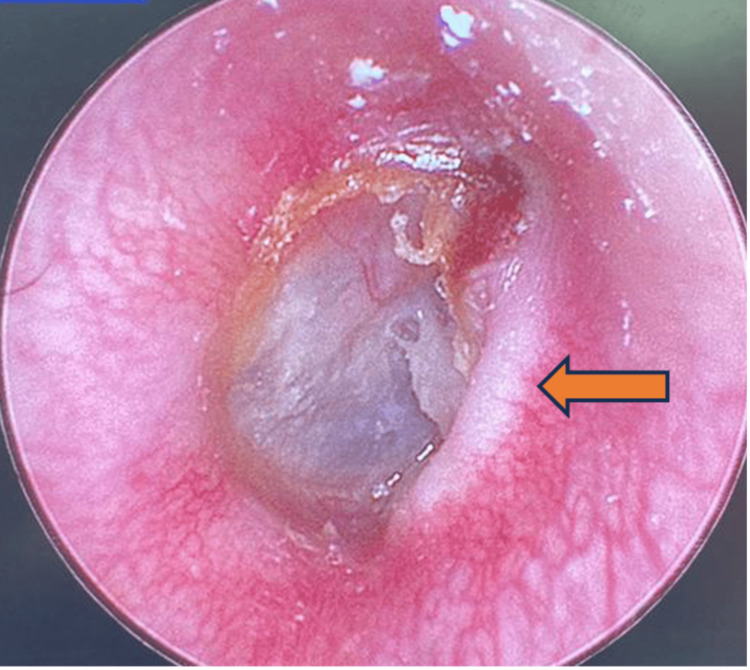
Otoscopic examination of the right ear shows soft tissue prolapse with indentation at the anterior wall of EAC close to the TMJ region upon opening the mouth (arrow).

Examination of the face shows right-sided facial fullness from the zygoma to the upper neck with crepitus. Otherwise, it was non-tender, no skin changes, and the facial nerve was intact. Oral cavity examination showed that the lower jaw shifted to the left upon mouth opening and back to normal upon closing with a loud audible crepitus sound on the right TMJ. A fluid-like crepitus sound was heard on auscultation. Otherwise, the mouth opening is not limited. The nose examination was unremarkable. Otoscopic examination of the right ear shows soft tissue protrusion at the anterior wall of the external auditory canal (EAC) close to the TMJ region upon closing of the mouth (Figure 1) and prolapse with indentation upon mouth opening (Figure 2).

There was no perforation at the tympanic membrane. The left EAC and the tympanic membrane were normal. Flexible nasopharyngeal laryngeal cope was unremarkable. An audiometric test showed right mild to moderate mixed hearing loss and left mild sensory neural hearing loss. The patient refused admission for further management. In view of the patient having an ongoing infection to the right ear, oral cefuroxime 500 mg BD for one week with aural advice was instigated.

**Figure 3:**
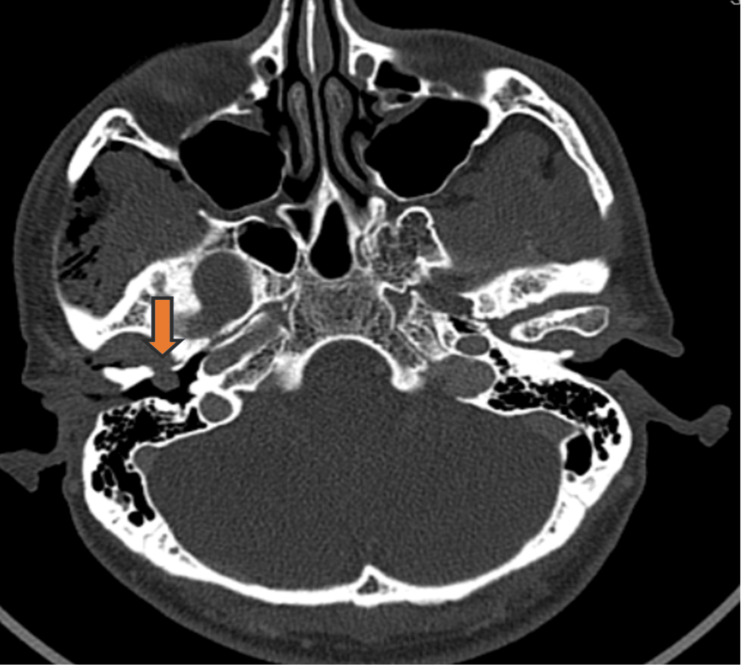
Contrast enhances computer tomography of the neck showed a bony defect at the anterior wall of the medial one-third of the right bony portion of the external auditory canal (EAC). The bony defect measures 0.6x0.7 cm (width x height) (arrow).

Contrast-enhanced computer tomography of the neck showed a bony defect at the anterior wall of the medial one-third of the right bony portion of the EAC. The bony defect measures 0.6x0.7 cm (width x height) (Figure 3).

**Figure 4:**
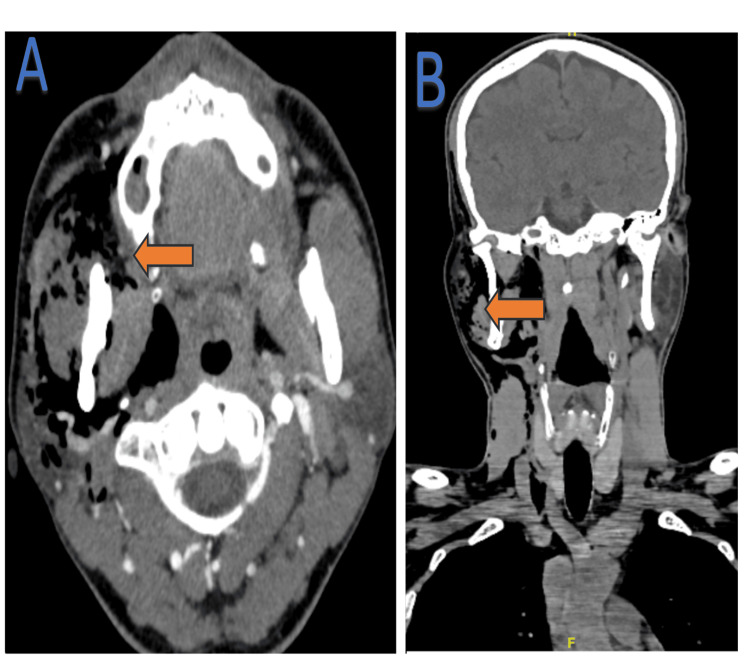
Extensive subcutaneous emphysema was noted in the right masticatory spaces, including the right masticator, buccal, parotid, parapharyngeal, and pharyngeal mucosal, right submandibular, right anterior and posterior cervical spaces and up to the right anterior chest wall. A: Axial view; B: Coronal view

At the site of this bony defect, there was a prolapse of soft tissue arising from the right TMJ indicating fistulous communication between the right EAC and right TMJ. The condyle of the right mandible was intact without periosteal reaction or cortical erosion. Extensive subcutaneous emphysema was noted in the right masticatory spaces, including the right masticator, buccal, parotid, parapharyngeal, and pharyngeal mucosal, right submandibular, right anterior, and posterior cervical spaces and up to the right anterior chest wall (Figure 4).

**Figure 5:**
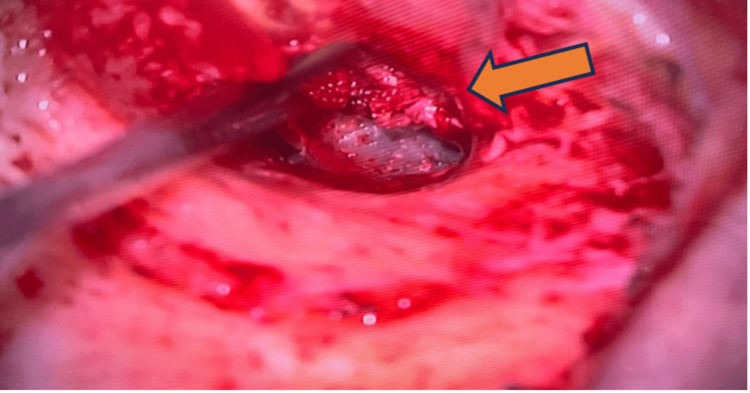
Granulation tissue occupying medial one-third of the EAC, which is attached to the anterior wall and tympanic membrane (TM) (arrow).

A week later, the facial and neck subcutaneous emphysema had resolved; however, a crackling sound at the right ear upon chewing persisted, which was disturbing to the patient. Due to that reason, we decided to proceed with surgical intervention. During surgery, we had difficulty and limited access to the defect via an endoscopic approach. Therefore, a combined endoscopic-assisted and open repair via a preauricular approach was opted. Intraoperative findings showed granulation tissue occupying the medial one-third of EAC, which is attached to the anterior wall and tympanic membrane (TM) (Figure 5).

**Figure 6:**
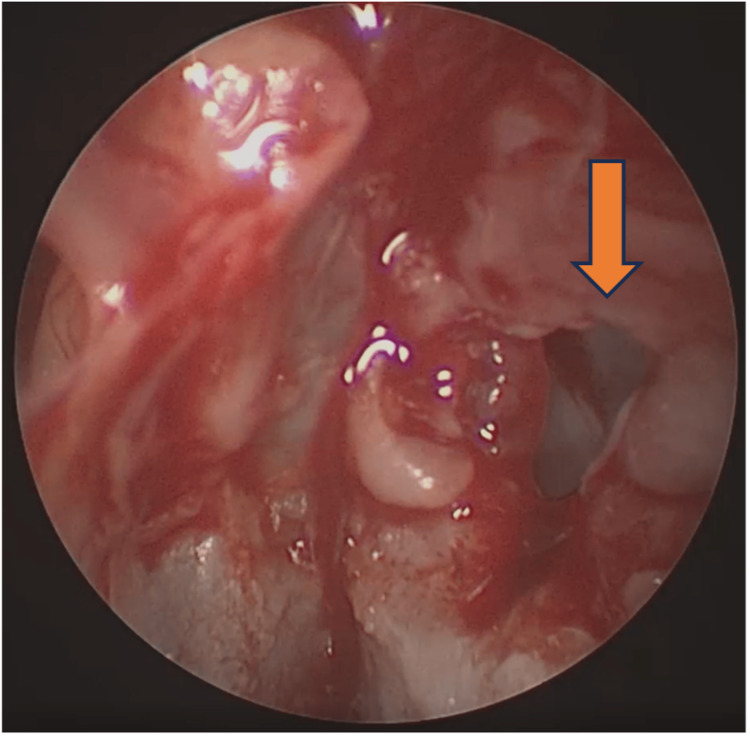
The defect was seen with fistulous communication between the right EAC and TMJ (arrow).

A bony defect was seen close to the TM. Additionally, there is a fistula seen communicating the EAC with TMJ. A preauricular incision was made, and the anterior-inferior portion of the tympanic bone was freed of soft tissue. A defect was visible, and a full border was identified (Figure 6).

**Figure 7:**
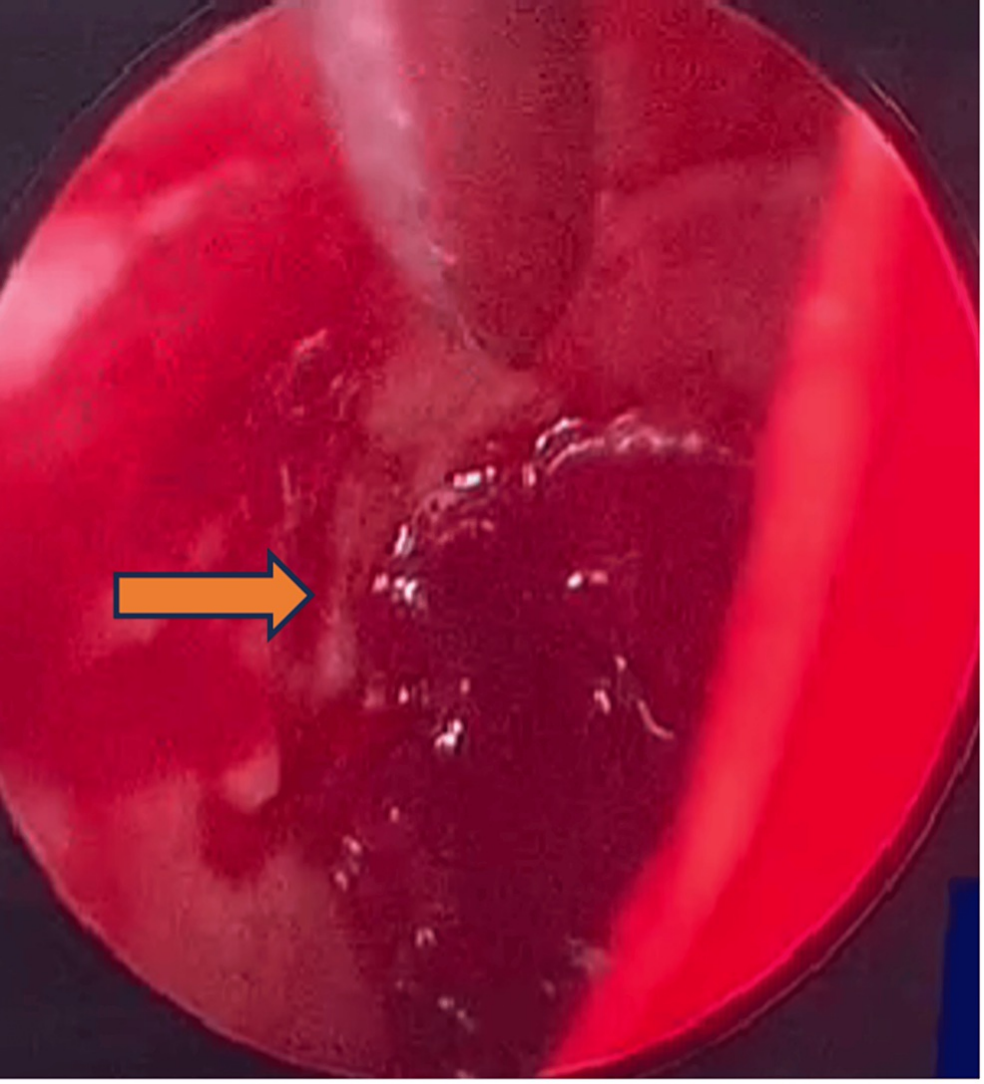
The cartilage allograft was positioned between the defect and the temporomandibular joint (TMJ) with the perichondrium facing the TMJ (arrow).

A tragal cartilage graft with perichondrium was harvested to reconstruct the auditory canal. The cartilage allograft was positioned between the defect and the TMJ with the perichondrium facing to the TMJ (Figure 7). The auditory canal was filled with BIPP and antibiotic-infused gauze for seven days following surgery and changed twice weekly.

**Figure 8:**
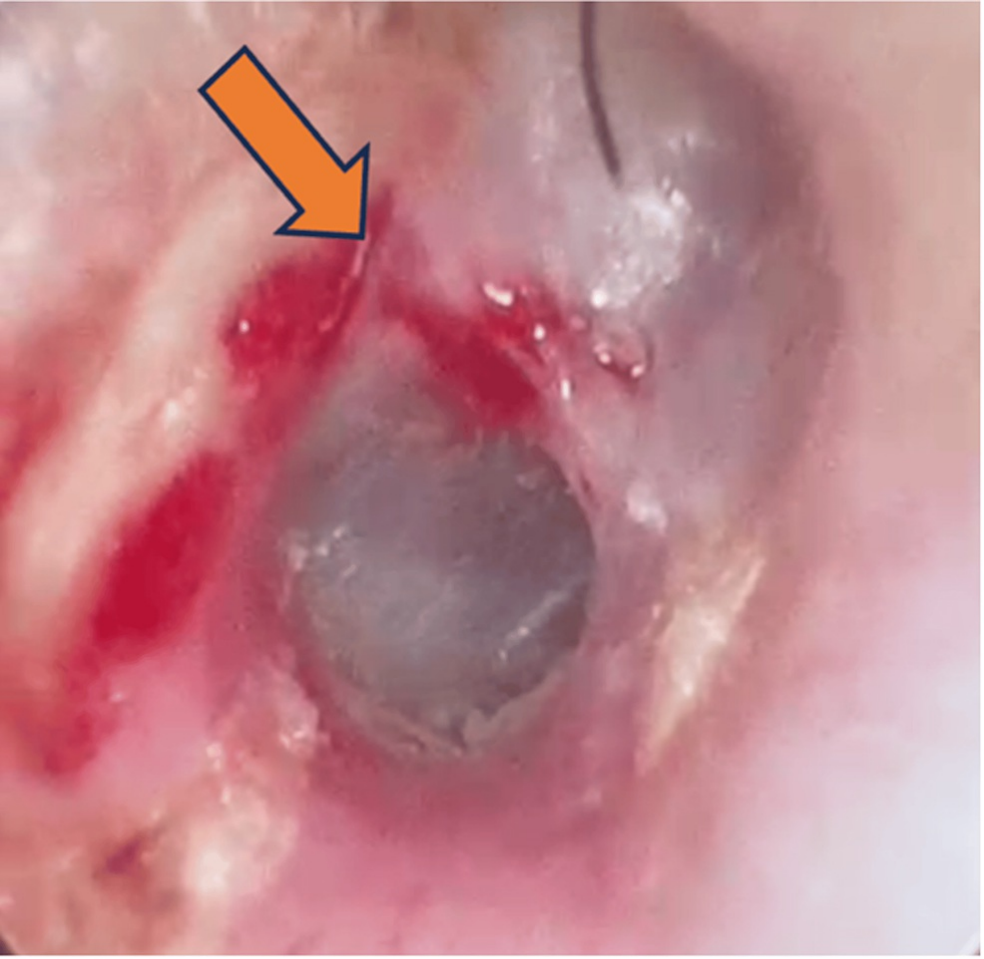
Two months post-op, there is a synechia extending from the anterior wall to the posterior wall of the EAC resulting in the narrowing of the bony portion of the ear canal (arrow).

Two months postoperatively, the patient develops synechia extending from the anterior wall to the posterior wall of the EAC, resulting in a narrowing of the bony portion of the ear canal (Figure 8). This patient complained of ear fullness in the right ear. However, the initial concern regarding the clicking sound has already been rectified. In addition, limited indentation was observed when clenching but not when opening and closing the mouth when speaking.

## Discussion

Herniation of TMJ into the EAC is rare. A cadaveric study revealed a 7.2% incidence of the persistent foramen of Huschke [3]. A defect in the bony auditory canal can result in the prolapse or herniation of the TMJ tissue. Anatomically, the EAC is closely associated with the TMJ, and it is separated from it by a thin bony anterior wall [4]. Furthermore, it increases the likelihood of protruding the TMJ complex into the EAC as the distance between the TMJ complex and EAC becomes shorter when the mouth is closed.

The causes of the anterior wall of EAC defect can be because of trauma, chronic infection, tumour erosion, iatrogenesis, and a congenital defect of the tympanic bone [5]. In our case, the latter was the most probable cause as the patient denied a history of trauma, neither previous history of tumour nor previous surgical intervention. Congenital defect of the tympanic bone leads to persistent foramen of Huschke [1]. At birth, the tympanic bone is in the form of a U-shape, which has anterior and posterior prominence and is usually closed by the age of five. However, defective membranous ossification leads to congenital anterior wall defect [1].

Persistent foramen of Huschke is usually asymptomatic and may develop symptoms at an older age [3]. A study concluded that mastication-induced stress to the EAC bone defect may result in the softening of the intervening tissues or expansion of the bony defect, leading to TMJ tissue herniation [3]. This helps explain the reason our patient with congenital abnormalities does not have symptoms until they reach maturity/later age. 

In addition to otalgia, otorrhea, tinnitus, and mandibular joint pain [3,6-7], uncommonly cervicofacial subcutaneous emphysema may also be one of the patient's presenting complaints [5]. Aside from our case, we are aware of only one other example of self-induced subcutaneous cervicofacial emphysema linked with an EAC lesion [5].

Clinically, palpable subcutaneous emphysema is an invasion of air into the subcutaneous layer of the skin [5]. Factors leading to cervicofacial subcutaneous emphysema can be categorized according to three different routes of action: cutaneous, such as open tracheostomy; mucosal, such as during facial fracture; and alveolar membranes, such as during asthma and bacterial infection [8]. Our patient had a preexisting anterior wall defect induced by years of chronic subluxation from eating and speaking, resulting in mucosal thinning. Once an infection develops, the mucosa may become fragile and torn easily, resulting in the formation of a fistula. The fistula causes air to become trapped in the extracapsular tissue, resulting in negative pressure within the joint space as the condyle moves anteriorly during mouth opening.

Rarely can a spontaneous fistula form between the anterior wall of the EAC and the TMJ. The defect of the anteroinferior portion of the tympanic bone, also known as the foramen of Huschke, is hypothesised to be the origin of this uncommon ailment. Other possible reasons include infections such as otitis externa. There are two potential causes of fistula formation in our case: a long-standing defect and a history of ear infections. In addition, head-and-neck radiation, mandibular condyles or otologic fractures, and TMJ surgery may all contribute to this incidence [6].

A simple dynamic otoscopic examination is sufficient to diagnose TMJ herniation [9]. While the patient's mouth is closed during an examination, there is a protrusion of soft tissue mass at the anterior wall of the EAC, which prolapses upon the mouth opening [4]. This perfectly matches the definition of a hernia, as it is also the case in this instance. To plan for surgical intervention, contrast-enhanced computed tomography helps detect osseous defects, the size and location of the herniation, and the surgical approach [10]. Patients with TMJ herniation are known to have more severe defects than those with anterior wall defects but no herniation. Remarkably, TMJ protrusion has also happened through a minor defect, and it is hypothesised that mechanical stress from mastication, in addition to the size of the defect, may play a role [4].

Treatment for anterior wall defect depends on the aetiology and severity, which can be either conservative or surgical repair [4]. A radiological study concluded that only 0.4% presented with TMJ soft tissue herniation out of 13.4% who had persistent anterior wall defect upon reviewing a 1025 temporal bone computer tomography (CT) [1]. Nakasato et al. reported that their patient underwent reconstruction of anterior EAC defect using composite graft, auricular cartilage, and temporalis fascia with fibrin glue and showed no recurrence after 20 months of follow-up [9]. Furthermore, in a report described by Anand et al., a polypropylene implant was utilized and secured with a titanium miniplate over the zygomatic arch, using a preauricular technique by Al-Kayat and Brawley. They conclude that polypropylene is effective and safe for treating such situations based on the report's favorable outcome [2]. In another study by Cho et al., a patient with subcutaneous emphysema and no inflammatory symptoms responded favourably to an injection of hyaluronic acid into the affected TMJ [5]. Additionally, Crombie et al. proposed an alternative conservative treatment involving an aural toilet and a mandibular advancement splint to prevent the condylar head from moving backwards and striking the anterior EAC [7]. In this case, we initially attempted to repair the defect using a transcanal approach; however, because of the difficulty in visualizing the medial margin of the defect, with it being located very close to the margin of the tympanic sulcus, we opted for an open approach via the preauricular region, and, after establishing the location of the defect, we position the tragal cartilage between the defect and the TMJ with the perichondrium facing toward the TMJ. This method allows us to utilize autologous tissue from the same incision site, which is advantageous. Moriyama et al. performed a similar approach in which autologous tragal cartilage was used and repaired with fibrin glue. The EAC was then packed with gauze soaked in Achromycin olive Vaseline and removed 13 days postoperatively. Five years after surgery, there was no indication of a recurrence [3].

## Conclusions

Foramen of Huschke is a bone defect in the tympanic plate that can lead to spontaneous herniation of the temporomandibular joint into the external auditory canal. Otitis externa seldom causes cervicofacial subcutaneous emphysema with underlying anomalies of the bony section of EAC's anterior wall. This case illustrates one of these complexities. CECT is the gold standard for diagnosis and therapy facilitation. Depending on the patient's condition, conservative or surgical intervention may be recommended.
